# Orthographic Visualisation Induced Brain Activations in a Chronic Poststroke Global Aphasia with Dissociation between Oral and Written Expression

**DOI:** 10.1155/2019/8425914

**Published:** 2019-07-02

**Authors:** Jurgita Usinskiene, Michael Mouthon, Chrisovalandou Martins Gaytanidis, Agnes Toscanelli, Jean-Marie Annoni

**Affiliations:** ^1^National Cancer Institute; Faculty of Medicine, Vilnius University, Vilnius, Lithuania; ^2^Neurology Unit, Department of Neuro and Movement Sciences, Faculty of Science and Medicine, Fribourg University, Switzerland; ^3^Fribourg Cantonal Hospital, Switzerland; ^4^Cabinet de Logopédie du Sonnenberg, Fribourg, Switzerland

## Abstract

We propose a method of orthographic visualisation strategy in a poststroke severe aphasia person with dissociation between oral and written expression. fMRI results suggest that such strategy may induce the engagement of alternative nonlanguage networks and visual representations may help improving oral output. This choice of rehabilitation method can be based on the remaining capacities and, therefore, on written language. Most notably, no study so far addressed how orthographic visualisation strategy during speech rehabilitation might influence clinical outcomes in nonfluent aphasia and apraxia patients.

## 1. Introduction

While many studies have examined poststroke functional reorganization of language brain networks in aphasic patients with left hemispheric damage [1, 2], the behavioural and functional effects of teaching patients to implement new compensatory cognitive strategies in their language impairments remain underexplored [3]. Such speech therapies can indeed be very effective through spontaneously evolved suboptimal strategies [4]. For example, speech and language therapeutic strategies shown to improve recovery of identifying an object or entity [5] may include teaching the patient to utilize visual recognition (access to structural description), use semantic representation, learn pronunciation (phonologic representation), and employ motor planning (articulation) [6]. It is typically as reported in chronic left hemispheric aphasic patients who correctly identify a subject involving left IFG, left TG, SMA, and right IFG [7, 8]. Verbally identifying objects has also been demonstrated to be mediated by a brain network independent of the classical network [9].

## 2. Materials and Methods

The present study focuses on an 18-month rehabilitation program of patients' ability to correctly name objects in a rare case of chronic severe nonfluent aphasia with severe apraxia of speech (AOS) following a large left hemisphere stroke. The therapy consisted of training articulatory movements using visual support, then teaching the patient to visualise the orthographic form of the word before pronunciation. The strategy suggested to the patient was to retrieve simultaneously the visual mental representations of the articulatory movement for a given phoneme, as well as the corresponding grapheme; then, the orthography of the word allowed to deconstruct the words into phonemes that would be subsequently enunciated [10]. To investigate the functional consequences of this therapeutic approach, we recorded at the end of therapy fMRI the poststroke participant performing a task of naming objects from their pictures with success measured by correct application of the taught orthographic visualisation technique. We hypothesised that using the visual word imagery strategy might help access to phonological/phonetic stores for allowing word production [11]. At the brain functional level, we predicted that the orthographic strategy could be associated with nontraditional language areas which had been destroyed in the patients in our study. We also expected activations related to visual form processing cortex activation due to orthographic word visualisation strategy.

### 2.1. Case Report

**Figure 1:**
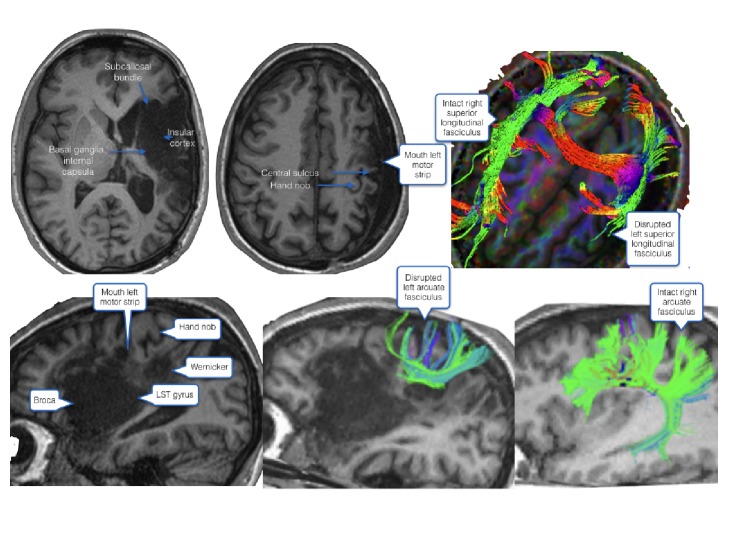
Poststroke brain lesions are shown in 3T MRI.

A 29-year-old female (T.W.), a native French speaker, suffered from global aphasia and right hemiplegia following a left hemispheric stroke (NIHS 18/42), due to a dissection of the left internal carotid artery. The stroke involved the left cortico–subcortical frontotemporoparietal areas. Lesion included 44 – Broca's, 22 – Wernicke's, 4 – motor cortex areas, insular cortex, basal ganglia, the most medial and rostral portion of the subcallosal fasciculus, the periventricular white matter near the body of the lateral ventricle, deep to the lower motor/sensory cortex area for the mouth and disrupted left arcuate fasciculus (see Figure 1).

T.W. was a governess by profession, right-handed, and an active smoker. For 9 to 12 months following the stroke, she was practically mute and could not pronounce a single word. On first language examination two weeks after the stroke, she was diagnosed with global aphasia with AOS, buccofacial apraxia and oral suppression, agraphia, oral and written comprehension impairment, and executive dysfunction. She underwent intensive inpatient neurorehabilitation for four months then was discharged with ambulatory speech therapy, physiotherapy, and ergotherapy. She started to walk in the city (with a stick) accompanied by her family and her boyfriend; she also resumed listening to music and watching movies. However, the severe AOS remained. Nine months after the stroke, she was still nearly mute, with no oral functional output, only the capacity to repeat some isolated phonemes* (/a/, /ou/, /m/, /f/, /n) *and syllables (*mamama, nanana, moumoumou*), and could whisper “no”. She had necessitated an explicit control for every phoneme output. However, her written production improved and she started to write words and small sentences with her left hand. T.W. communicated essentially by gestures during the first months. At home, she began to use some written support to communicate, either with a pencil in her left hand or with a speech program named I-Word Q, which allowed her to write. She could also use a selected written vocabulary on her iPad. Nine months after the stroke, despite some residual difficulties in finding written words, she could take advantage of her written communication ability for her daily messages, particularly with smartphone messages. Language and neuropsychological performances of T.W. at the different postonset times and steps of speech rehabilitation are demonstrated in Table 1. The following tests were acquired: (1) for language evaluation – Montréal-Toulouse 86 (MT-86) [12]; (2) for description of a picture – Nicholas & Brookshire (1993) [13]; (3) for writing – Croisiles battery [14]; (4) for associative gnosia – Columbia's b Test [15], (5) for memory – 5 mots de Dubois [16], (6) for executive functions – BREF (Batterie rapide d'evaluation frontale de Dubois ‘The Frontal Assessment Battery') [17]. Total score = 18; cut-off = 15. Oral expression was still very severely impaired. She could repeat 10/18 isolated phonemes, and 9/19 isolated syllables. Word and nonwords repetition was not possible due to AOS. She started to repeat some disyllabic trained words although this did not appear on the formal evaluation. Oral naming was still impossible due to AOS and inability to initiate a phonation. Reading of words and nonwords was still impossible (Table 1, 2nd Column). The written expression had much better recovered at 9 months after stroke. Particularly, written naming performance improved from 60/144 at 3 months to 90/144(62%) at 9 months. There was no significant frequency effect (high-frequency words: 49/72; low-frequency words: 41/72, chi^2^ is 1.89, p <.17), but a length effect was present (46/60 for 1 syllable words, 39/60 for 2 syllables words, 5/24 for 3 syllables words, chi^2^=23, p < 0.001). In writing to dictation, she was able to write 64% of the presented items with neither regularity effect, nor significant frequency effect (Croisile's battery: 9/10 high-frequency words, 5/9 medium frequency words, and 4/9 low-frequency words, chi^2^ = 4.7, p <.094). Concerning the error types in written production, she could write the first syllable, but could not always complete the word. Particularly, she helped herself with the vocalisation of some isolated syllables, but could not always vocalise or respect the order of the syllables in the word. There were mainly literal paragraphs (omission, substitution of letters) and nonphonologically plausible errors (examples: “canic” for “canary”; “abomas” for “abdomen”) unless some random words were correctly written (e.g., “oignon”, “bapteme”). At the receptive and semantic levels, word comprehension was relatively preserved in the oral (8 correct word to image association /9) and the written (4/5) domain. T.W. partially preserved her ability to correctly classify in the semantic field 86% of the presented words in an intracategorical designation test. The score at the pyramid and palm tree test visual version had been 47/52 (lower limit) 3 months after stroke.

Her performances in the written and the oral domain at 9 months after stroke suggested preserved input (preserved writing to dictation) and semantic processing (satisfactory performance in oral and written comprehension). The results at our images designation test and the image pyramid and palm tree test also indicated that the general domain semantic system is preserved. The AOS was present in all oral outputs (naming, reading, repetition) for both words and nonwords in the oral domain sparing only isolated phonemes. Written expression was partially spared, but showed some nonphonological errors affecting words and nonwords, with a length effect but no consistent regularity and frequency. The whole pattern pointed to a locus of impairment mostly at the postlexical impairment, at the phonetic-phonological level in the oral domain, and a partially preserved writing ability with some milder graphemic buffer type of dysfunction. The difficulties in sentences comprehension were possibly syntactic.

**Figure 2:**
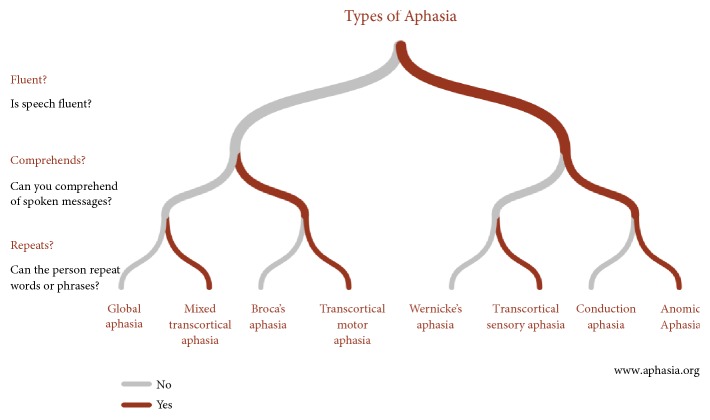
General types of aphasia (from https://www.aphasia.org).

T.W.'s initial global aphasia evolved at 9 months in a severe Broca's aphasia with severe postlexical oral impairment at the phonetic-phonological level leading to massive AOS. Interestingly, her writing was much better preserved despite moderate impairment at the graphemic buffer level. Some syntactic difficulties were also present. Main types of aphasia in general are presented in Figure 2. Aphasia evolvement in T.W. is presented in Table 2.

### 2.2. Speech Therapy

One year after the onset, given the dissociation between impossible oral and relatively preserved written expression, T.W. was instructed in the use of visual word imagery strategy by the language therapist (A.T.). The goals of the therapy were focused on (1) improving orthographic-visual skills to reinforce T.W.'s expertise to transform a set of letters into a visual word form; (2) memorising written form of the word based on a neurolinguistic programming (NLP) technique to automate the visualisation strategy adding eventually upward eye movements [18]; (3) training of the visualisation (integration of the written word's picture, then visual mental rehearsal) so that T.W. could also improve written words production. The speech therapist observed the link between “visual orthographic imagery” of the word and its successful translation in articulatory movements. However, if the sound produced aloud was not congruent with the internal graphemic representation of the word, it immediately blocked its oral production. Thus, at the early stage, a “visualisation technique” was implemented to “reprogram” the articulation of phonemes. For each phoneme, a pictogram representing the correct articulatory movement was associated with the corresponding grapheme, and the set was memorised visually. This visualisation was cued using principles of NLP (increasing the capacity of imaging language representation by coinitiating eye movements towards up) for the 32 phonemes of the language [18]. Then T.W. focused her visualisation of mouth movements at the level of the syllables. Then T.W. was given as homework a sequence of pictograms corresponding to a certain number of basic words, as well as the written form of these words. She trained daily visually to memorise the succession of articulatory movements with this corresponding word (NLP visualisation technique [19], to develop progressively the oral form of the word and thus a “basic” oral lexicon). Support to construct the verbalisation of a word was through the simultaneous activation of the word visual orthographic image and the visible mental representations of the articulatory movements. Gradually, this visual stimulus enabled her to exploit initial lexical potential. T.W. was able to follow this new therapy program and could integrate it into her word finding strategies. To facilitate word visualisation, she developed eye movement techniques used by healthy subjects and aphasic persons in NLP approach. T.W. explained her personal method to find and articulate a word: “*I move my eyes in certain directions to facilitate certain aspects of language. First, I position my eyes downwards: in this way I know what I want to mean, but cannot yet articulate the words. Then, I lift my eyes towards up to see the written word, and I find it more “natural” to pronounce, particularly for simple words. For more difficult words, I block my mouth and stay silent until I have seen the written word. It is a way I have trained with the speech therapist*”.

The therapy lasted 18 months, twice a week for one hour sessions. After six months of this new technique, her spontaneous speech improved with a few comprehensible words, and after 18 months she could construct short sentences with a decreased number of phonetic and phonemic errors. She reported that using the visualisation technique of the written word gave her a more precise pronunciation. However, at the end of the therapy, she said that she used the visualisation technique less frequently and that the words were found easier also without visualisation techniques. The posttherapy language evaluation still revealed moderate-to-severe Broca's aphasia (nonfluent speech, but right comprehension) with AOS, phonetic and phonemic paraphasias, agraphia, mild oral-facial-lingual apraxia, dyscalculia and executive dysfunction – difficulty in inhibition and motor planning.

On posttherapy formal evaluation, she could name correctly 20 out of 31 items of the MT 86 battery in comparison to 0 at 9 months after stroke. Repetition and reading improved: 12 words out of 25 and 4 nonwords out of 5. She could also read aloud correctly 15 words out of 25 and 2 nonwords out of 5. Fluency for oral description of animals improved, but letter fluency remained impaired (see Table 1).

### 2.3. Experimental Design

At the end of therapy picture naming fMRI was recorded. Three fMRI experimental conditions were presented (see Table 3): (1) explicitly naming the picture activating the visual representation of the written word (cueing condition); (2) naming the picture and suppressing this cueing; (3) naming the picture by reading the written word below the picture (control condition). In total, 144 different pictures were presented in 3 separate experimental runs of 48 stimuli. Each run lasted 7 minutes and was composed of the three blocks of 16 pictures each: (i) one block in which T.W. explicitly used the cueing, (ii) one block of naming with suppressing cueing, and (iii) one control condition. Each picture lasted 6 seconds on the screen. Between the pictures, a fixation cross was displayed with a variable duration of 1.5-3 second. The position of the pictures was identical between conditions (centre of the screen) but with different colours on the border of the screen to help to recognise the task. The background colour around the black-white pictures was grey for each condition. Before the scan, a training session outside the scanner was performed to familiarise participants with the experimental design. The black and white line drawings were selected from the picture database of Cycowicz et al. [20]. The three lists of pictures (see Tables 3 and 4) were matched for frequency, name agreement, and visual complexity [21]. Moreover, the lists were matched by phonological neighbourhood density and syllabic structure, as these factors can have a strong effect on language production errors in aphasic patients [22, 23]. Grapheme-to-phoneme correspondence could play an important role on whether the words can be sounded out, a skill that is being encouraged by visualisation. So, we categorised the regularity of each word according to the following criteria: regular words: words with a one-to-one grapheme-phoneme correspondence or words with one-to-one grapheme-phoneme correspondence with a silent grapheme in a terminal position. All other words were defined as irregular. Kruskal Wallis test revealed that the word lists were matched for regularity. These items were not part of the therapy, although they may have been used in free communication training. The E-Prime 2 software (Psychology Software Tools, Inc.) was used for stimulus presentation.

MRI was carried out with Siemens 3T Prisma at the “Centre d'Imagerie BioMédicale” (CIBM, https://www.cibm.ch) of the University Hospital of Geneva. T1-weighted images were acquired with an MPRAGE sequence, voxel size: 0.5x0.5x1mm, number of sagittal slices: 176, TR/TE/TI=2300/2.27/900 ms, flip angle = 9°. Functional T2*∗*weighted Echo Planar images with blood oxygenation level-dependent (BOLD) contrast were acquired with: voxel size: 2x2x3.5mm, 29 ascending axial slices, interslice spacing = 0.35mm, TR/TE=2000/30ms, Flip angle=85°. Diffusion Tensor Imaging (DTI) data was acquired using Echo Planar images with a voxel size of 1.6x1.6x1.6mm, 74 axial slices, TR/TE=3500/60ms, 64 noncollinear directions with b-value=1000 s/mm^2^, 4 min duration.

### 2.4. Data Analysis

Data for the output in verbally identifying pictures was audiotaped and then scored by nomenclature accuracy and errors by a speech-language pathologist (C.G.M.) trained in clinical output analysis. She rated the first answer, except in cases where T.W. produced just a phoneme and then the correct answer (there were only 2 such productions). Since the errors were effectively complicated to decide between phonemic and phonetic, we clustered them in a single group, classifying them as segmental errors [24]. We defined the errors as follows:

- No response: no answer or just phoneme or unintelligible production.
- Semantic error: the semantic link between production and the target word.
- Segmental error: the phonological or phonetic transformation of the structure of the word, e.g., substitution, omission, addition, or transposition of phonemes/segments and also phonetic distortions [24–26].
- Neologism: unrecognisable word, more than 50% transformation.
- Mixed: segmental + semantics.

fMRI images were analysed with SPM12 software (Welcome Trust Centre for Neuroimaging, Institute of Neurology, University College London). Unwarping, spatial realignment, slice timing, normalisation on the Montreal Neurological Institute (MNI) space with 2x2x2mm^3^ voxel size, and smoothing with a Gaussian kernel of 6-mm full-width-at-half-maximum (FWHM) were used for preprocessing [27]. The preprocessed volumes of T.W. and the control (healthy volunteer) were submitted to a fixed-effect analysis with movement parameters included as regressors of no interest. We studied the difference between fMRI picture naming tasks in both participants. A statistical threshold of p<0.001 at the voxel level, corrected for multiple comparisons with an extended cluster threshold size of 100 contiguous voxels (p_cluster_< 0.05; family-wise error (FWE) corrected). Anatomical locations were checked with the neuromorphometrics probabilistic atlas provided in SPM12. Dynamic causal modelling (DCM) analysis was carried out with SPM12 using a mask from the interaction contrast between T.W. and control. We defined a region of interest (ROI) of 6 mm, centred inside the highest significantly activated clusters in T.W compared to the control. T.W.'s DCM model for picture naming was then studied firstly using orthographic visualisation priming (called strategy) and then without visualisation priming (called nonstrategy) in the second condition. We compared connection values between two ROIs in each condition.

## 3. Results

### 3.1. Picture Naming Performance

There was no significant difference of T.W.'s spoken correct responses across conditions (see in Table 5). The number of correct responses was between 33% and 45% in the three sessions. We did not find a difference between the different conditions in terms of errors. The vast majority of the errors was of segmental nature (phonetic-phonological type) in all conditions, confirming the evolution of aphasia. Some errors showed mixed pattern, e.g., for “violin” she said /gEtar/ (guitare = semantic paraphasie + “gEtar” instead of “guitar”, so that was a segmental error). Simple syllable structure words were named 37/79, and complex structure words 23/65 (chi^2^ statistic is 1.9, p value 0.165) and this difference was insignificant in all conditions.

### 3.2. fMRI Results

**Figure 3:**
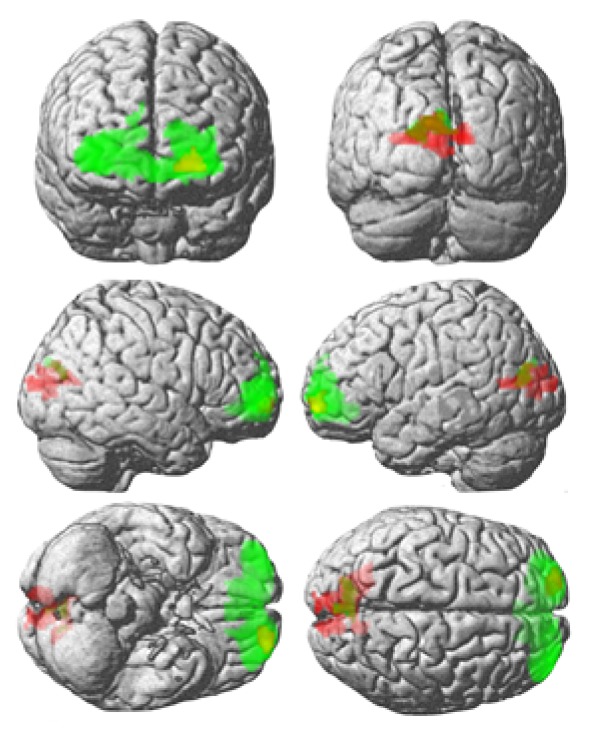
Brain activation during the picture naming with orthographic visualisation priming relative to naming without priming in red and relative to reading in green, as well as the superposition of both in yellow (p=0.01, cluster size≥100).

**Figure 4:**
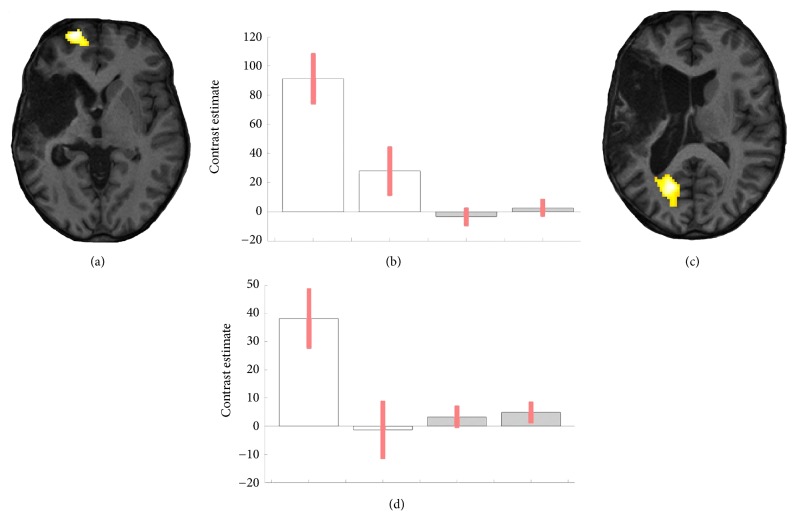
Interaction contrast (a, c) and 90% C.I. contrast estimates (b, d) between the patient (white bars) and the control (grey bars) during the picture naming with orthographic strategy (first and third bars) relative to naming without strategy (second and fourth bars). Cluster x y z coordinates (mm) in (a), (b): -18 -68 12, in (c), (d): -20 62 -4 (p=0.01, cluster size≥100).

**Figure 5:**
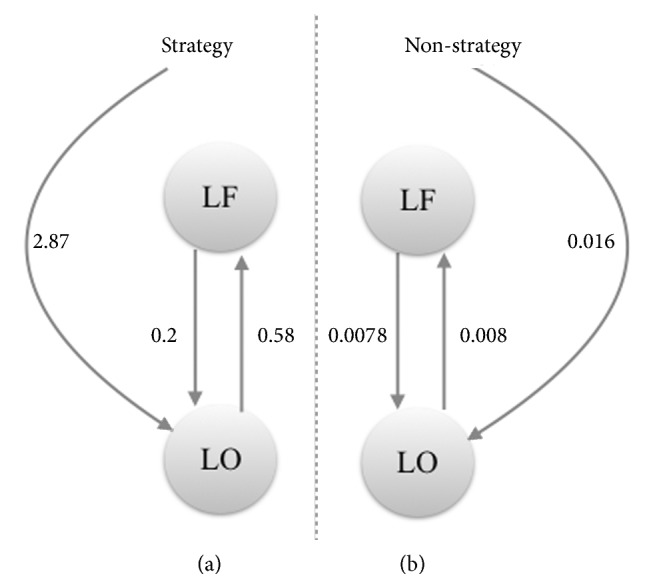
DCM model shows the strength (presented in numbers) and direction (arrows) of functional connectivity between two regions of interest: left frontal (LF) and left occipital (LO) during strategy in comparison to nonstrategy.

Naming visual pictures with an orthographic imagery prime in contrast with naming pictures without priming activated the left calcarine cortex (coordinates mm x y z: -14 -70 12, cluster size 609, p_FWE  corr_ = 0.000) in T.W. brain. Naming visual pictures with an orthographic prime in contrast with reading activated the left and right superior and middle frontal gyrus (-24 60 -4; 34 54 -4, 40 52 2, cluster size 2787, p_FWE  corr_ = 0.000) and left cuneus and left superior occipital gyrus (-12 – 76 20, -20 -78 16, -4 -86 28, cluster size 154, p_FWE  corr_ = 0.039) in T.W. Picture naming without priming relative to reading activated the left medial frontal cortex and anterior cingulate gyrus in T.W. (-6 56 -10; -4 38 -6; 16 36 -4, cluster size 390, p_FWE  corr_ = 0.000). T.W. brain activations during the picture naming with an orthographic visualisation prime were relative to naming without priming and relative to reading, as well as the superposition of both (see Figure 3). No significant occipital activations were found in T.W. for picture naming without priming contrasted with reading or the opposite. For the interaction contrast, there were significant activations in the left calcarine cortex and cuneus (-18 -68 12;-8 -80 24;-14 -78 16; cluster size 207, p_FWE  corr_ = 0.004) and left superior and middle frontal cortex (-20 60 -4;-18 58 4;-32 -58 2; cluster size 202, p_FWE  corr_ = 0.004) in comparison to the difference in orthographic visualisation priming versus without priming contrast between T.W. and the neurotypical control. Significant results of this interaction are presented in Figure 4. Based on the DCM model, which compared the priming (strategy) and without priming (no strategy) effects in T.W.'s second session, we found a priming effect on the strength of the functional connectivity between two ROIs—the left occipital and prefrontal. The strength of the connection between these two areas was higher during the priming in comparison without priming (see Figure 5).

## 4. Discussion

We reported an exceptional case of severe Broca's aphasia and AOS with a strong dissociation between severe mutism due to postlexical severe phonetic-phonological impairment and a partially preserved written ability, in a poststroke young female with largely affected Broca's, Wernicke's, motor, insular, subcortical areas, and interrupting the arcuate fasciculus.

Nine-month poststroke evaluation confirmed severe postlexical oral impairment at the phonetic-phonological level leading to massive AOS and functional writing despite moderate graphemic buffer dysfunction.

We could also demonstrate an improvement in the speech output in a severely chronic aphasic person (T.W.), using a speech therapy technique characterised by written words visualisation and mental training of articulatory movements.

By visualising written words during naming and word finding tasks, T.W. could regain oral expression of the word and correct the severe AOS. Functionally, we tried to mimic therapy sessions in an experiment where we asked the patient to use her visual strategy applying the visual priming explicitly. This priming condition, compared with control and without priming, resulted in the relative increase in brain activity in left visual and frontal areas.

Concerning the aphasiological case description, this is an intriguing case of dissociation between absent oral expression and preserved written expression. A similar case was described earlier after a lesion of the third frontal gyrus and the upper temporal gyrus [28]. The authors suggested that the rupture of the audio-phonological loop (with degradation of the verbal working, memory) made this dysfunction worse. However, in their case, Cambier et al. described no apraxia of speech, but an involuntary production of utterances. In the classical analyses of 500 aphasic patients, Basso et al. described 7 cases of selective impairment of speech. Three cases were with “pure anarthria,” two patients with anarthria in the context of Broca's aphasia, and two other aphasic patients with fluent aphasia except in writing [29]. Particularly, the two cases of Broca aphasia were characterised at the oral level by the absence of verbal production, clinical signs of “phonetic disintegration”, very little speech outflow, and strenuous articulation and partially preserved (although not intact) writing abilities. This dissociation was longstanding in one patient (14 months). The lesion was not known, but both patients were young (< 35 yrs old), had less than 9 years of schooling, and suffered from right hemiplegia. One case also exhibited some problems in sentences (syntactic) comprehensions, as in our study. In their anatomic-clinical study, Kreisler et al. found that mutism depended on large front-putaminal lesions: “a finding consistent with the anatomy of severe Broca's and transcortical motor apraxia”; impaired repetition associated with insula-external capsule lesions, and paraphasia on external capsule lesions [30]. T.W.'s stroke involved frontal areas including Broca's, motor and insular cortex and basal ganglia. Moreover, T.W. had also a lesion of premotor cortex, which is known to cause AOS [31]. It has been confirmed that persistence of AOS after 12 months is associated with large left hemispheric stroke and strokes that involve Broca's area [32]. In our case, the dorsal parietal-frontal stream of the language (pre- and postcentral gyri, left superior temporal and angular, the insula and Broca area) were affected. There was a complete disruption of the left arcuate fasciculus. Thus, we suggest that massive stroke involvement may explain the severe segmental (phonological-phonetic) impairment.

Nevertheless comprehension recovery was impressive. An initial reason for the retention of comprehension ability may be linked to the patient's young age. It has been reported that younger patients with equivalent lesions tend to have more Broca's aphasia pattern [33] and less comprehension deficit [34]. A second reason may be that the deficit was more syntactic than phonological or semantic, as shown by the good words and poor sentence comprehension. Thirdly, we suggest that the use of written imagery more easily compensated for the phonological impairment, not only in expression, but also in comprehension.

Concerning rehabilitation, to the best of our knowledge, our report is the first to describe in an aphasic patient the behavioural and brain changes associated with using visual imagery of the written words to name the objects in pictures and their appropriate verbalisation. The occipital cortex was previously reported to be involved in the ability to name correctly pictorial representations in people with aphasia [35]. Picture naming has also been shown to be mediated by a brain network independent of the classical arcuate fasciculus [9], which was destroyed in our poststroke participant. T.W.'s adoption of visual word imagery strategy was initially implemented to improve spelling through orthographic retraining [36].

Our findings were partly correlated to previous evidence that relying on visual speech perception for the treatment of nonfluent aphasia could improve speech production^,^ possibly through activation of speech motor automatism through different channels [37]. In our study, there was no increase in occipital activations for reading as compared to picture naming without cueing, suggesting that visual form related activity was not higher for reading than for picture naming. In contrast, visual cortices activations were higher when T.W. used orthography visualisation for naming. Whether this pattern could be explained by previous evidence that orthographic processing in neurotypical adults is supported by the functional integration of visual processing, articulation and semantics remain to be elucidated with clinical group studies [38].

The superior frontal gyrus (SFG) has been involved in a wide range of functions such as cognitive control, execution, and motor control [39]. T.W.'s engagement of left prefrontal region and superior frontal gyrus could explain that for access to image orthography of the pictures she was using a phonological buffer. Because most of T.W.'s language network was damaged (the left arcuate fasciculus, Wernicke's and Broca's areas were impaired), T.W. necessarily used alternative pathways to support the interactions between an occipital prefrontal region and to improve articulation planning. Although it was not confirmed when compared to the control subject, an engagement of the right anterior structures in the case of imaging may have had a role in this left occipital-frontal activation. Adaptive plasticity of the right homologous area during speech production has been repeatedly shown to contribute to recovery in certain circumstances [40]. Our results suggest that visual word imagery in naming is here associated with increased left occipital activation, which indirectly increases the left prefrontal network. Such activation pattern is proposed to be mediated by a nonclassical linguistic network which plays a role in speech output.

Recovery of speech output may depend on several factors, such as the amount of spared tissue in the left inferior frontal gyrus (LIFG) [41], damage to Broca's area, and treatment associated with the recruitment of areas in the left hemisphere [42]. The integrity of the left superior longitudinal fasciculus (SFL) [43] and the poststroke rehabilitation time [44] play an important role as well. Despite the complete necrosis of the LIFG and severely damaged left white matter tracts (especially SLF), T.W.'s speech output using an orthographic visualisation strategy has improved. The late stage at which the imaging strategy was introduced into rehabilitation suggests an indirect effect of this approach rather than a spontaneous recovery. Since there is no control case for T.W., one could argue that the visualisation technique used in the therapy is not necessarily the cause of the patient's verbal improvement. In fact, intensive training could also play an important role in the articulatory improvement of T.W. Indeed, speech therapist reported that after 1 month of the absence of therapy, e.g., during summer holidays, T.W. had more difficulties in pronouncing words. However, in this situation, T.W. had more difficulty to both visualise words and in motor movement. Access to visual support, in general, became less natural, and so communication. This required a “reexplanation” of the “strategies” allowing visual representation of articulatory movements, the spelling of words and verbal production.

No effect of syllabic complexity in word production was present, independently of the conditions. This absence of effect in postlexical impairment may be unexpected. However, it has been shown in another aphasic that it is possible to improve production of complex items with therapy [45]. It is possible that this absence of difference is related to the intensive therapy oriented towards orthographic imagery.

Based on fMRI findings, we postulate that T.W. was accessing word retrieval through the visual pathways and improved articulation planning using an adopted visual word imaging technique. More specifically, we postulate that the process of imagining the written words activated visual-spatial representations along the right sided dorsal route and modulated brain activity in the left occipital and left frontal regions.

Our study suffers several limitations. First, fMRI recording is challenging the aphasic participant, due to uncontrolled movements and AOS. To minimise movement effects, we immobilised T.W.'s head and applied unwarping during the postprocessing of the fMRI data. Also, we included the movement parameter of regressor of no interest in the statistical model for the patient and control. We also familiarised both participants with the task before image acquisition. Second, we did not carry out a full neuropsychological language evaluation and fMRI before the applied strategy, and therefore we cannot report any differences between the baseline and therapy-induced brain activation. Finally, we cannot exclude that the succession strategic and nonstrategic influenced the data. However, the mixing of conditions would have been too exhausting for T.W. The experiment lasted only 7 minutes, but fatigue may affect the accuracy of the 2nd and 3rd conditions. Moreover, it is possible that some of the prefrontal cortex activations are due to dealing with a novel task and this is less obvious, as the testing continues. Despite these limitations, T.W.'s data suggest that the visual imaging component of the orthographic strategy modulated intact left frontal region through occipital activation.

## 5. Conclusions

We suggest that visual imagery techniques in a rehabilitation setting may activate alternative nonlanguage networks and may improve articulation of the word in chronic aphasic individuals. This choice of rehabilitation methods can thus be logically based on the remaining capacities and, in this case, on a partially preserved capacity in written language.

## Figures and Tables

**Table 1:** Language and neuropsychological performance.

Time after stroke	36 months	9 months	3 months

Period of therapy	18 months	pretherapy	pretherapy

*Oral production*			
Series	15/17	0*∗*/10	0*∗*/10
Description of a picture	nonfluent agrammatism	mute apraxia	mute apraxia
Pictures Naming	20/31	0*∗*/31	0*∗*/31
Verbal fluency (1')			
Letter *«* s *»*	3	0*∗*	0*∗*
Category *«* animals *»*	13	0*∗*	0*∗*
*Oral Repetition*			
Words	12/25	0*∗*/25	0*∗*/25
Nonwords	4/5	0*∗*/5	0*∗*/5
Sentences	0/3	0*∗*/3	0*∗*/3
*Oral comprehension *			
Words	9/9	8/9	9/9
Sentences	29/38	24/38	11/15*∗∗*
*Reading aloud*			
Words	15/25	0*∗*/25	0*∗*/25
Nonwords	2/5	0*∗*/5	0*∗*/5
Sentences	0/3	0*∗*/3	0*∗*/3
*Written Naming *			
Words	NA	90/144	60/144
*Writing to dictation (left hand)*			
Name, last name, address	OK	OK	OK
Words	16/ 24*∗∗*	19/28	7/18*∗∗*
*Written comprehension *			
Words	4/5	4/5	5/5
Sentences	7/8	5/8	5/8
*Praxia (Gestures)*			
Buccofacial	11/13	5/7	not done
Symbolic gestures	4/5	3/5	1/4
Using objects gestures	9/10	7/10	0/4
No signification gestures (unimanual)	3/3	3/3	3/3
*Calculation *	1/5	0*∗*/5	1/5
Multiple choice answer	8/8	7/8	not done
*Others*			
Memory Dubois 5 words	10/10	not done	not done
Executive functions (FAB)	11/18	9/18	0/12

0*∗*: no production because of speech suppression due to apraxia of speech. *∗∗*: simplified version used in this case.

**Table 2:** Aphasia evolvement.

Time poststroke	initial	36 months
*Types of aphasia*	*Global*	*Broca's*
Speech	mute	non fluent
Comprehension	impaired	preserved
Repetition	impaired	effortful
Motor signs	right hemiplegia	right hemiparesis, buccofacial apraxia

**Table 3:** Experimental design, picture naming fMRI tasks.

(I) Cueing condition	(II) Suppressing the cueing	(III) Reading condition
abeille, aigle, aiguille, ampoule, ane, avion, balai, baleine, bras, bus, cactus, canard, chaise, chenille, ciseau, cle, coeur, coq, echelle, elephant, escargot, etoile, gant, girafe, hibou, jumelles, lampe, lion, maison, montagne, mouton, nez, ours, parapluie, peigne, piano, pinceau, pomme, prise, regle, renard, rhinoceros, sifflet, singe, tomate, tracteur, vache, voiture	araignee, arbre, arrosoir, autruche, bague, balle, banc, bougie, brosse, canape, carotte, cerise, chaussure, chemise, cheval, cheveux, chien, citron, cloche, commode, cravate, croissant, dauphin, douche, feu, fleche, fourchette, fourmi, fraise, lit, main, oeil, oiseau, oreille, panier, pasteque, pied, pipe, plume, poisson, requin, souris, table, tigre, tortue, trompette, violon, vis	ananas, banane, botte, boussole, bouteille, bouton, ceinture, cerf, cerveau, champignon, chat, chaussette, chauvesouris, cheminee, citrouille, corde, crabe, crayon, cuillere, cygne, ecureuil, enveloppe, louche, loup, montre, mouche, orteil, papillon, pingouin, poire, fleur, hache, jambe, kangourou, lapin, livre, louche, loup, montre, mouche, orteil, papillon, pingouin, poire, porte, pouce, poule, raisin, roue, scie, soleil, squelette, tournevis, velo, verre, zebre

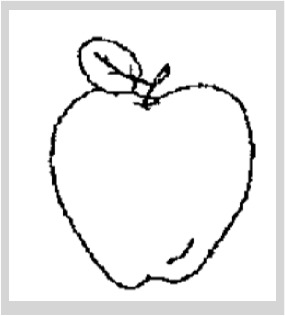	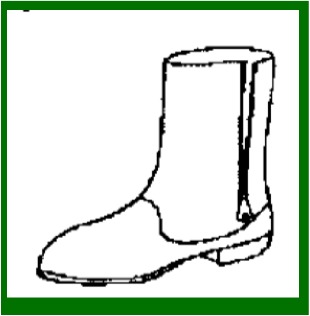	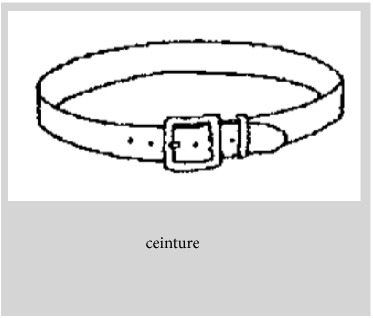

**Table 4:** Statistics of the word list in three conditions for the fMRI picture naming task.

Conditions	Frequency	Name agreement	Visual Complexity	Phonological neighborhood density	Syllable Structure	Regularity
	*Mean*					

*I Cueing*	43.97	95.77	2.97	8.60	1.37	0.58
*II Suppressing*	40.55	96.06	3.01	9.12	1.54	0.60
*III Reading*	27.21	95.83	2.88	9.83	1.43	0.72
		*p value*				
*I vs II*	843	841	833	775	103	837
*I vs III*	310	966	681	522	537	135
*II vs III*	248	882	480	721	312	197

**Table 5:** Evaluation of T.W. naming production.

	Correct out of 48 items	No response	Semantic	Segmental	Neologism	Mixed
Naming with priming	n 17	n 6	n 5	n 15	n 3	n 2
Naming without priming	n 22	n 0	n 2	n 16	n 3	n 5
Reading	n 21	n 2	n 0	n 22	n 3	n 0

## Data Availability

The main steps of the demonstrations for each result are clearly reported in the text and the article is fully consistent without the support of any additional data.
